# Infectivity of Symptomatic Malaria Patients to *Anopheles farauti* Colony Mosquitoes in Papua New Guinea

**DOI:** 10.3389/fcimb.2021.771233

**Published:** 2021-12-22

**Authors:** Lincoln Timinao, Rebecca Vinit, Michelle Katusele, Tamarah Koleala, Elma Nate, Cyrille Czeher, Thomas R. Burkot, Louis Schofield, Ingrid Felger, Ivo Mueller, Moses Laman, Leanne J. Robinson, Stephan Karl

**Affiliations:** ^1^ Vector-borne Diseases Unit, Papua New Guinea Institute of Medical Research, Madang, Papua New Guinea; ^2^ Australian Institute of Tropical Health and Medicine, James Cook University, Smithfield, QLD, Australia; ^3^ Molecular Diagnostics Unit, Swiss Tropical and Public Health Institute, Basel, Switzerland; ^4^ Department Biozentrum, University of Basel, Basel, Switzerland; ^5^ Population Health and Immunity Division, Walter and Eliza Hall Institute of Medical Research, Melbourne, VIC, Australia; ^6^ Department of Medical Biology, The University of Melbourne, Melbourne, VIC, Australia; ^7^ Malaria Parasites and Hosts Unit, Department of Parasites & Insect Vectors, Institut Pasteur, Paris, France; ^8^ Vector-Borne Diseases and Tropical Public Health Division, Burnet Institute, Melbourne, VIC, Australia

**Keywords:** direct membrane feeding assay, *Plasmodium vivax*, *Plasmodium falciparum*, Papua New Guinea, *Anopheles farauti*, mosquitoes

## Abstract

*Plasmodium* transmission from humans to mosquitoes is an understudied bottleneck in the transmission of malaria. Direct membrane feeding assays (DMFA) allow detailed malaria transmission studies from humans to mosquitoes. Especially for *Plasmodium vivax*, which cannot be cultured long-term under laboratory conditions, implementation of DMFAs requires proximity to *P. vivax* endemic areas. In this study, we investigated the infectivity of symptomatic *Plasmodium* infections to *Anopheles farauti* colony mosquitoes in Papua New Guinea (PNG). A total of 182 DMFAs were performed with venous blood collected from rapid diagnostic test (RDT) positive symptomatic malaria patients and subsequently analysed by light microscopy and quantitative real time polymerase chain reaction (qPCR). DMFAs resulted in mosquito infections in 20.9% (38/182) of cases. By light microscopy and qPCR, 10 – 11% of *P. falciparum* and 32 – 44% of *P. vivax* positive individuals infected *An. farauti*. Fifty-eight percent of *P. vivax* and 15% of *P. falciparum* gametocytaemic infections infected *An farauti*.

## Introduction

Transmission between the human host and the mosquito vector is a crucial step in the malaria parasite life cycle. It represents a bottleneck where parasite numbers shrink from millions in the human body to less than a hundred in the mosquito vector (Smith et al., 2014). Transmission through the mosquito is thus vulnerable to interruption and is a key focus of malaria research (Churcher et al., 2015; Sauerwein and Bousema, 2015), with research tools including membrane feeding assays designed to explore this transitioning phase of the parasite. DMFAs were initially developed by Rutledge and colleagues in 1964 in which malaria parasites were exposed to mosquitoes *via* a membrane feeding apparatus (Rutledge et al., 1964).

Direct Membrane Feeding Assays provide a means to investigate the still poorly understood process of human to mosquito transmission and the resulting mosquito infection. For example, DMFAs can be used to study the infectiousness of different human malaria reservoirs, and estimate their contribution towards transmission (Graves et al., 1988; Diallo et al., 2008). This can include symptomatic, patent infections as in the present study and asymptomatic, often low-density infections (Kiattibutr et al., 2017). In addition, DMFAs can be used to study the effect of drugs, vaccine candidates and immune factors on the development of the mosquito stages of the *Plasmodium* parasites (Bousema et al., 2012; Delves et al., 2012; Sattabongkot et al., 2015; Vallejo et al., 2016). Also, DMFAs provide an opportunity for circumventing some of the operational and ethical complicating factors associated with feeding mosquitoes directly on the skin of malaria infected individuals. Finally, there is evidence that there is no clustering of gametocytes in the skin as initially perceived thus making DMFAs a reliable tool for infection studies (Meibalan et al., 2019; Talman et al., 2020).

Despite these advantages, DMFAs are resource intensive, require an insectary and rely on stringent logistics for sample collection, handling, rapid transportation and processing as it has been shown that the time between blood collection and performance of the DMFA can impact assay outcome, most likely due to premature gametocyte activation (Churcher et al., 2012; Sattabongkot et al., 2015; Soumare et al., 2021). As a further complication, conducting DMFAs with *P. vivax* requires proximity to endemic areas in order to access infected samples as continuous culture of this parasite species remains elusive (Roobsoong et al., 2015). Papua New Guinea (PNG) is amongst the countries with the highest *P. vivax* burden in the world, thus *P. vivax* is a research priority for the country and infected blood samples can still easily be obtained (Cattani et al., 1986; Müller et al., 2003; Howes et al., 2016; World Health Organization, 2019; World Health Organization, 2020). Establishing DMFAs with *P. vivax* provides a tool to study *P. vivax* transmission that is of potentially global relevance.

DMFAs were performed in PNG previously in 1983 - 1985 in village-based malaria surveys, prior to diagnosis and on known gametocyte carriers in clinical outpatient populations in Madang and Goroka (Graves et al., 1988). In the present study, we investigated the infectivity of blood samples obtained from symptomatic, rapid diagnostic test (RDT)-positive individuals to *Anopheles farauti* colony mosquitoes.

## Materials and Methods

### Sample Collection

This study was conducted at the PNG Institute of Medical Research (PNGIMR) in Madang Province, PNG, between May 2014 and November 2018. Study participants were recruited from Madang Town Clinic and Yagaum Rural Health Centre. Ethical approval was received from the PNGIMR Institutional Review Board (IRB #1516) and the PNG Medical Research Advisory Committee (MRAC #16.01). Written informed consent was received from all individuals enrolled in the study. Individuals presenting with malaria symptoms were tested with a malaria rapid diagnostic tests (RDT). In the present study, CareStart Malaria Pf/PAN (HRP2/pLDH) Ag Combo RDTs kits (Access Bio, Cat No. RMRM-02571CB) were used. From RDT-positive individuals venous blood samples (3 - 5 mL) were collected in vacutainers which contain spray-coated lithium heparin (BD, North Ryde, NSW, Australia) and immediately stored in a beverage cooler flask (Coleman Company Inc, Kansas, USA) filled with warm water (~37.0°C, measured by a digital thermometer attached to the flask). We also measured their Hemoglobin level using a HemoCue machine (HemoCue ^®^, Mt Waverley, VIC, Australia), their temperature using a digital thermometer and their weight using a bathroom scale.

In the present study, the time between sampling and feeding was approximately 20 - 30 min for samples collected at Yagaum clinic, located in a 10 min walking distance from the insectary. Transport of blood samples collected in Madang town clinic took about 1.5 - 2 h and involved a 30 - 40 min drive.

### Mosquito Colony Maintenance, Membrane Feeding Assays, and Mosquito Dissection

The present study used an *An. farauti sensu stricto* colony, which was first adapted in Rabaul, East New Britain province of PNG in 1968. In 1984 females from Agan village, Madang were added to the Rabaul colony in an attempt to back cross. The colony was subsequently used in several studies (Sweeney, 1987; Graves et al., 1988; Beebe et al., 2000; Collins et al., 2002). The colony was maintained using established methods (Nace et al., 2004). To conduct DMFAs, 3 - 5 day old female mosquitoes were put into paper cups (50 -100 per cup). One mL of human blood sample was inserted into the water jacketed glass feeder where we initially exposed up to 400 mosquitoes with 100 mosquitoes per cup (X 4 cups) and then changed with up to 200 with 50 mosquitoes per cup (X 4 cups) as it was less crowded and the feeding rate was much improved (Timinao et al., 2021).

The feeding cups and water jacketed glass feeder were set up in the laboratory prior to the arrival of blood samples to minimise the time between blood sample collection and direct membrane feeding. The light in the insectary was dimmed and the glass feeder with the cups were covered with a dark cloth for the period of feeding. After ~15 - 20 min, the cups of *An. farauti* were removed from under the glass feeders and any unfed mosquitoes were removed. The cups containing the fully fed mosquitoes were kept for 7 - 9 days before dissecting for oocysts (Ouedraogo et al., 2013; Sattabongkot et al., 2015). Dissection was performed as described elsewhere (Ouédraogo et al., 2013). Briefly, mosquito guts were stained with 0.2% mercurochrome for 10 - 15 min and oocysts were counted under a light microscope at 10 X magnification. Oocysts per midgut were counted once by an experienced microscopist.

### Light Microscopy and PCR Detection of Malaria Parasites

Retrospective diagnosis of the malaria parasites was performed by light microscopy and quantitative real-time PCR (qPCR). Thick and thin blood films were prepared using standard WHO methodology. The blood films were stained with 4% Giemsa stain for 30 min (World Health Organization, 2010). Slides were read according to WHO standards and by WHO certified microscopists. Parasite density was calculated by multiplying the parasite count/200 WBC count (or 500 WBC count if the parasite count is <100) by 8000 leukocytes (World Health Organization, 2010). The final parasite density was calculated by taking the geometric mean of the densities obtained from reads by two expert microscopists. Discrepancies in the presence or absence of parasites, parasite density (i.e. if they differed by a factor of 10) and parasite species between the two microscopists was resolved by a third expert microscopist. DNA extraction was performed on 250µL of red blood cell pellets using Favogen DNA extraction kits (Favogen Biotech Corp, Ping Tung, Taiwan) and performed according to the protocol for extraction of genomic DNA from blood. Following DNA extraction, a qPCR assay was performed to quantify the infection and determined the parasite species as described elsewhere (Wampfler et al., 2013). Briefly, this is a probe based qPCR assay where a conserved region of the 18SrRNA gene was amplified for both *P. falciparum* and *P. vivax*.

### Statistical Analyses

Prism 6.01 (GraphPad Software, La Jolla, CA USA) and Stata 13 (StataCorp, College Station, TX, USA) were used to analyse data. To compare proportions, two-sample tests of proportions were used. To test the influence of a continuous variable (such as parasite density) on a binary outcome variable (such as DMFA success rate), logistic regression was used. To test the association between two continuous variables such as infection rate in the successfully infected mosquitoes versus gametocyte density we used non-parametric correlation analysis (Spearman’s rank correlation). 

## Results

### Study Population

Selection of patients relied on RDT diagnosis. Subsequent light microscopy examinations of the corresponding blood slides and molecular diagnosis by qPCR were conducted for 182 RDT-positive participants. A total of 45 patients were recruited from Madang town clinic while 137 were recruited from Yagaum clinic. **Table 1** shows the characteristics of the study population and **Table 2** shows the results from RDT, light microscopy examination and molecular diagnosis by qPCR.

**Table 1:** Characteristics of the study population.

Demography	Median (range) or n/N (%)
Age in years (n=182^a^)	17 (5-55)
Female (n=182)	91/182 (50.0%)
Weight, kg, (n=175^b^)	47 (14-96)
Hemoglobin, g/dl, (n=118^b^)	9.1 (4.2-13.7)
Temperature, °C, (n=161^b^)	36.6 (34.1-40)
Fever, >37.5°C, (n=161)	48/161 (29.8%)

a Eight individuals with unknown age so were considered as adults.

b These data were not collected from all 182 patients. Values are presented as proportions (n/N) and percentage or median and range.

**Table 2:** RDT, Microscopy and qPCR diagnosis of malaria parasites.

Diagnosis		n	n/N (%)	95% CI
**RDT**	HRP2	55	30.2	23.65 - 37.45
	pLDH	37	20.3	14.74 - 26.92
	HRP2 & pLDH	90	49.5	41.97 - 56.95
**Microscopy**	*P. falciparum* asexual only	68	37.4	23.65 - 37.45
	*P. falciparum* asexual with gametocytes	20	11.0	6.84 - 16.46
	*P. vivax* asexual only	28	15.4	10.47 - 21.46
	*P. vivax* asexual with gametocytes	24	13.2	8.63 - 18.98
	*P.falciparum* with gametocytes & *P.vivax* with gametocytes	2	1.1	0.13 - 3.91
	*P.falciparum* asexual & *P.vivax* with gametocytes	1	0.5	0.01 - 3.02
	Microscopy negative	37	21.4	15.70 - 28.11
**qPCR**	*P. falciparum*	80	44.0	36.62 - 51.49
	*P. vivax*	55	30.2	23.65 - 37.45
	*P.falciparum* & *P.vivax* mix	20	11.0	6.84 - 16.46
	PCR negative	27	14.8	10.01 - 20.85

Results of the diagnosis by RDT, microscopy and qPCR, N=182 samples in each case.

### Malaria Diagnosis

The largest proportion of individuals (49.5%; 95% CI 41.97 - 56.95%) was RDT positive for both, HRP2 and pLDH tests while 30.2% (95% CI 23.65 - 37.45%) and 20.3% (95% CI 14.74 - 26.92%) of patients were positive only for HRP2 or pLDH-based tests, respectively. Light microscopy revealed that the largest proportion of symptomatic patients in this study population were infected with *P. falciparum* (47.8%; 95% CI 40.90 - 55.86%) followed by *P. vivax* (28.6%; 95% CI 22.13 - 35.72). Median (range) parasite density was 6423 (110 – 51,040) parasites/µL for *P. falciparum* and 4240 (136–32,480) parasites/µL for *P. vivax.* There were 3 mixed infections (1.6%; 95% CI 0.3 - 4.74%) containing both, *P. falciparum* and *P. vivax*. The qPCR results revealed a slightly higher proportion of *P. falciparum* infections (40%; 95% CI 36.62 - 51.49%) than *P. vivax* infections (30.2%; 95% CI 23.65 - 37.45%). A higher proportion of the samples were diagnosed as mixed infections by qPCR as compared to microscopy (11% vs 1.6%). Over all we observed a higher proportion of patients who were diagnosed as *P. falciparum* positive by microscopy and qPCR as compared to RDT diagnosis (**Table 2**).

A total of 154/182 (85%) of the samples were concordant between qPCR and microscopy diagnosis. It was also observed that 19/182 (10.4%) patients were negative by microscopy but were positive by qPCR for malaria parasites while 9/182 (5%) were positive by microscopy but negative for qPCR (**Table 3**). A sensitivity of 94% was observed for qPCR diagnosis with a specificity of 49%. The positive predictive value (PPV) of 88% and a negative predictive value (NPV) of 67% was observed for qPCR. We also observed that there was no correlation between the microscopy diagnosis and qPCR for the following groups; *P. vivax, P. vivax* with gametocytes and *P. falciparum* with gametocytes. However, there was a significant but weak correlation observed with *P. falciparum* (Spearman’s rank correlation coefficient R=0.4, p<0.001).

**Table 3:** Comparison of malaria parasite detection in patients’ blood by microscopic examination and qPCR.

	Microscopy (Gold standard)			
	Test Result	+	–	Total
qPCR	+	136	19	155 (85%)
	–	9	18	27 (15%)
	Total	145 (80%)	37 (20%)	182 (100%)

### Mosquito Infection

**Figure 1:**
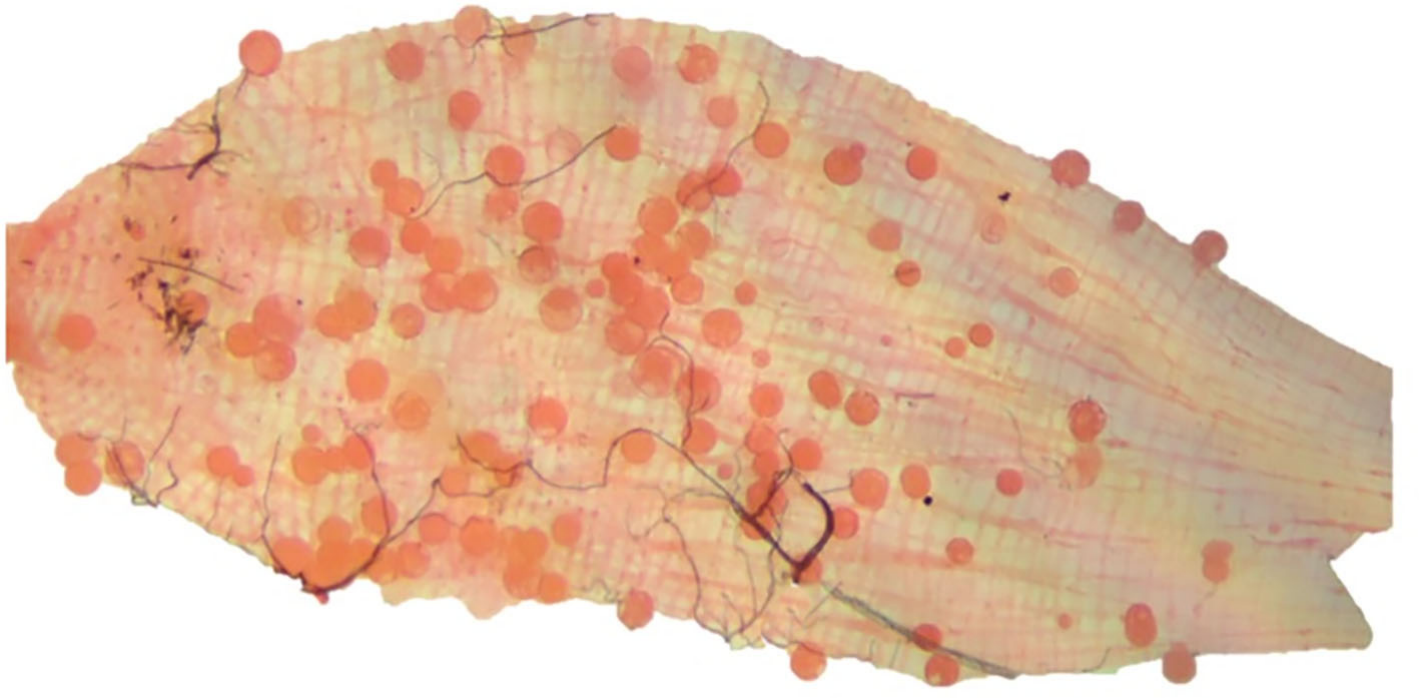
*P. vivax* infected midgut from *An. farauti* mosquito dissected in the present study. The image was taken on a Zeiss Primostar microscope equipped with an Axiocam 105 Color camera (Carl Zeiss Pty. Ltd.) at 10x magnification. The image was then edited using PowerPoint, Microsoft office 2010 and Adobe Photoshop CS6.

Overall, 38/182 (20.9%) of blood samples in DMFAs infected mosquitoes with 36/38 (94.7%) of the patients being recruited from Yagaum clinic while the remaining 2/38 (5.3%) were Madang town clinic. **Figure 1** shows an example of an *An. farauti* midgut infected with *P. vivax* oocysts 7 days post infection.

Although not significant we did observe a higher proportion of infections by individuals diagnosed with RDT as pLDH positive than those diagnosed as HRP2 positive (35.1% vs 27.3%, p=0.43) (**Table 4**). Interestingly, *P. vivax* infections diagnosed by light microscopy were significantly more infectious to mosquitoes compared to *P. falciparum* infections (44.2% vs. 11.4%, p<0.01). Similar observations were made with qPCR diagnosis (43.6% vs 10%, p<0.001). Within the *P. vivax* samples, a higher proportion of blood samples were infectious to mosquitoes when *P. vivax* gametocytes were detected by microscopy (58.3%). We noted that 32% (9/28) and 10.3% (7/68) of the *P. vivax* and *P. falciparum* infections that infected mosquitoes were gametocytaemic by microscopy. In addition, all the mixed infections (3/3) by microscopy gave rise to mosquito infections.

**Table 4:** Mean oocyst counts from DMFAs in *An. farauti* according to RDT, microscopy and qPCR.

RDT, microscopy & qPCR results	Proportion DMFAs resulting in mosquito infection			Proportion of mosquitoes infected^*^			Oocyst number
	n/N	% (95% CI)	n/N		% (95% CI)	average (range)	
RDT							
HRP2	15/55	27.3 (15.5 - 39.1)^a^	564/966		58.4 (55.2 - 61.5)	6 (1-106)	
pLDH	13/37	35.1 (19.7 - 50.5)^b^	349/863		40.4 (37.2 - 43.8)	27 (1-534)	
HRP2 & pLDH	10/90	11.1 (4.6 - 17.6)^c^	60/415		14.5 (11.2 - 18.2)	3 (1-17)	
Microscopy							
*Pf* asexual only	7/68	10.3 (3.1 - 17.6)^d^	66/376		17.6 (13.8 - 21.8)	5 (1-16)	
*Pf. +* gametocytes	3/20	15 (0 - 30.6*^§^*)	47/94		50.0 (39.5 - 60.5)	3 (1-9)	
*Pv.* asexual only	9/28	32.1 (14.8 - 49.3)^e^	218/627		34.8 (31 - 38.6)	9 (1-93)	
*Pv.* + gametocytes	14/24	58.3 (38.6 - 78)^f^	424/749		55.9 (52.8 - 60.2)	19 (1-534)	
*Pf. +* gametocytes *& Pv +* gametocytes	2/2	100 (NA)	13/69		18.8 (10.4 - 30.1)	3 (1-13)	
*Pf.* asexual only *& Pv* gametocytes	1/1	100 (NA)	83/89		93.3 (85.9 - 97.5)	7 (1-36)	
Microscopy Negative	2/39	5.1 (0 - 12*^§^*)	13/38		34.2 (19.6 - 51.4)	9 (1-29)	
qPCR							
*P. falciparum*	8/80	10 (3.4 - 16.6)	135/481		28 (24.1 - 32.3)	3 (1-43)	
*Pv.*	24/55	43.6 (30.5 - 56.7)	735/1501		49 (46.4 - 51.5)	12 (1-534)	
*P. falciaprum & P. vivax*	4/20	20 (2.5 - 37.5)	96/248		38.7 (32.6 - 45.1)	8 (1-106)	
*qPCR Negative*	2/27	7.4 (0 - 17.3)*^§^*	7/14		50.0 (23.0 – 77)	7 (1-24)	

Results of the diagnosis by RDT, microscopy and qPCR, N =182 while n = 38 in the successful infections.

^*^only infected mosquitoes were considered (i.e., uninfected mosquitoes were not included into this calculation); significant differences were observed in the proportions ^a^ vs. ^c^; ^b^ vs. ^c^, ^d^ vs. ^e^ and ^d^ vs. ^f^. No significant difference was observed between ^a^ vs. ^b^, p=0.43.

^§^95% confidence interval includes negative values.

All samples were collected from symptomatic RDT positive patients. Values are presented either as proportion (n/N) and percent, or as average and minimum to maximum range.

Pf., P. falciparum; Pv., P. vivax.

**Figure 2:**
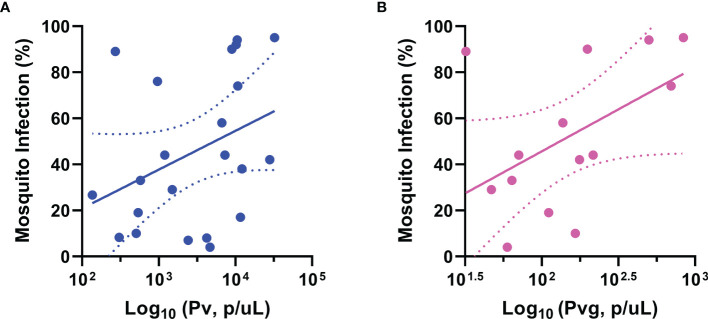
Correlation between mosquito infection rate and parasite burden of infected humans. Correlation between mosquito infection and **(A)** *P. vivax* parasites (R= 0.4, p=0.08), **(B)** *P. vivax* gametocytes (R= 0.6, p=0.04). The trend lines are linear regression while the area between the dotted lines represents the 95% confidence interval. Pv, *Plasmodium vivax*; Pvg, *P. vivax* gametocytes. Each dot represents a mosquito that was infected with 1 or more oocyst. **(A)** has 22 while **(B)** has 14 successful infections.

There was a weak correlation between the proportion of infected mosquitoes and *P. vivax* density by microscopy with the correlation approaching significance (p=0.08, Spearman’s rank correlation coefficient R = 0.4) as shown in **Figure 2A**. The proportion of infected mosquitoes was significantly correlated with *P. vivax* gametocyte density (p<0.05, Spearman’s rank correlation coefficient R=0.6) as shown in **Figure 2B**. However, the considerable scatter and correlation coefficient of R=0.6 indicated that the correlation was not very strong. There was no correlation between the mosquito infection rate and the copy numbers of *P. falciparum* or *P. vivax* by qPCR.

Infection success, i.e., DMFAs resulting in at least 1 infected mosquito, was not significantly correlated with parasite or gametocyte density when tested using logistic regression in any of these groups; *P. vivax*, *P. falciparum*, *P vivax* with gametocytes.

**Figure 3:**
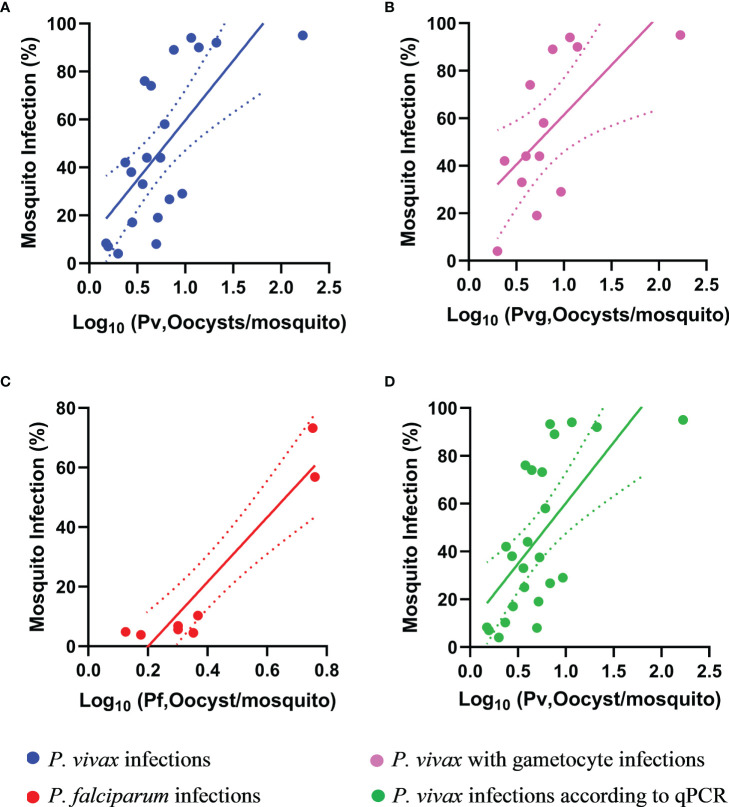
Correlation between proportion of infected mosquitoes and the mean oocyst number per infected mosquito. The oocysts per infected mosquito for **(A)** *P. vivax* (R = 0.7, p < 0.001), **(B)** *P. vivax* with gametocytes (R = 0.7, p < 0.01), **(C)** *P. falciparum* (R = 0.8, p < 0.05) and **(D)** *P. vivax* by qPCR (R = 0.7, p < 0.001). The trend lines are liner regression while the area between the dotted lines represents the 95% confidence interval. Pv, *Plasmodium vivax*; Pvg, *P. vivax* gametocytes; Pf, *P. falciparum*.

We observed moderate and significant correlations between the number of oocysts per infected mosquito midgut and the proportion of infected mosquitoes per DMFA according to microscopy diagnosis for the following; *P. vivax* (Spearman’s rank correlation coefficient R=0.7, p<0.0001), *P. vivax* with gametocytes (Spearman’s rank correlation coefficient R=0.7, p<0.01), and *P. falciparum* (Spearman’s rank correlation coefficient R= 0.7, p<0.05) as shown in **Figures 3A–C**. We also observed a moderate and significant correlation between oocysts per infected mosquitoes and mosquito infection by qPCR for *P. vivax* according to qPCR diagnosis (R=0.7, p<0.001) as shown in **Figure 3D**. There was no correlation observed between the proportion of infected mosquitoes and the copy numbers of *P. vivax* or *P. falciparum* by qPCR.

## Discussion

Currently the limitation with doing research with *P. vivax* is it is still difficult to maintain a continuous culture of *P. vivax*, which necessitates access to naturally acquired infections in field settings, often associated with additional cost and operational constraints. As such, a reliable *P. vivax* DMFA setup in an endemic setting can be of great value.

In the present study, we investigated the infectiousness of symptomatic, RDT-positive malaria cases obtained from local health facilities. In a resource constrained situation where diagnosis by microscopy is not readily available and where the primary diagnosis of malaria is performed by RDT, it is important to assess which RDT result will most likely lead to a mosquito infection. As commonly known, RDT results are not reliable in distinguishing between *Plasmodium* species in co-endemic settings, however, the present study shows that they can be used to prioritise samples selected for DMFAs to maximise the probability of a specific species being present in the sample and to increase infection success (**Table 2**) (World Health Organization, 2006). We observed that in the group of samples positive for only pLDH with the CareStart RDT the proportion of successful DMFAs was highest (35.1%) compared to HRP2 (27.3%) or when positive for both antigens (11.1%). This difference in proportions was statistically significant between pLDH and both antigens (p<0.01) but not between pLDH and HRP2 (p=0.43). In PNG where both *P. falciparum* and *P. vivax* are present in roughly equal proportions, *P. vivax* infections were more likely when the RDT is positive for only the pLDH antigen (World Health Organization, 2017). We therefore decided that by selecting samples only positive for pLDH over HRP2 (or both antigens) the likelihood of the sample being *P. vivax* would be significantly increased and DMFA success can be increased up to 3-fold. There is a sound biological explanation as to why acute *P. vivax* infections result in mosquito infections more frequently. It has been shown that *P. vivax* gametocytes develop faster, and are present and infectious at the onset of an infection while *P. falciparum* gametocytes can take 10 days to mature (Bousema and Drakeley, 2011). Consequently, lower infectiousness in symptomatic *P. falciparum* patients as compared to *P. vivax* patients is expected since people are likely to seek treatment before *P. falciparum* gametocytes have matured (Kiattibutr et al., 2017). Furthermore, HRP2 based RDTs can remain positive for 35-42 days after treatment and clearance of parasitaemia, while for pLDH it takes only 2 days before the antigen is cleared from circulation giving a more reliable result (Grandesso et al., 2016).

We observed that the proportion of samples that infect mosquitoes was higher for *P. vivax* (44.2%) compared to *P. falciparum* (11.4%) according to microscopy. Interestingly, we observed a higher mosquito infection rate (58.3%) for samples with *P. vivax* gametocytes detectable by light microscopy while the mosquito infection rate with *P. falciparum* gametocytes was low (15%). Although our findings are in contrast to what was observed previously by Graves and colleagues in *An. farauti* where they showed a 37.5% (6/16) infectivity with *P. vivax*, 18.8% (3/16) infectivity in *P. vivax* with gametocytes and a 48.1% (13/27) infectivity in *P. falciparum* with gametocytes, this may be due to the difference in sample sizes used (Graves et al., 1988). Our results show that our DMFA with *P. vivax* is about 4 times more successful than with *P. falciparum* especially when considering samples with gametocytes. Although we are uncertain as to why we observed low *P. falciparum* infections a possible explanation that we did not evaluate in this study is that immunity-related factors are responsible. This could be further studied by comparing, in parallel, DMFAs conducted with autologous plasma (i.e., replacement of patient’s plasma with the individual’s own plasma), whole blood (directly added to the feeder) and malaria-naïve plasma (i.e., replacement of patient plasma with plasma from a *P. falciparum* naive donor). Furthermore, a possible explanation is that this strain of mosquitoes may have become refractory to wild-type *P. falciparum* infection. This was observed with cultured gametocytes of *P. falciparum* which were fed to this strain of *An. farauti* mosquitoes *via* standard membrane feeding which resulted in an extremely low mosquito infection rate (Smith et al., 2014). This indicated that the *An. farauti* strain was refractory to cultured gametocytes and that could also be the case with wild-type parasites as well. Interestingly, our observations that this does not apply to *P. vivax* may be the basis for further studies into species-specific mechanisms of mosquito infection.

In the present study, we observed an 85% concordance between the microscopy diagnosis and qPCR diagnosis. We observed a 94% sensitivity and a 49% specificity when comparing qPCR with light microscopy as the reference method. This is a result of the lower limit of detection of the qPCR method, which is able to detect many more infections as compared to light microscopy. As qPCR is able to detect these sub-microscopic infections, the proportion of false positive is overestimated when compared to light microscopy leading to an apparently low specificity. The possibility of an infection (or no infection) by microscopy being confirmed by qPCR is expressed by a moderate PPV and NPV (88% and 67%). We note that the lack of having microscopy diagnosis being done prior to bleeding was a limitation in this study and light microscopy results were only obtained retrospectively by highly trained microscopists. We found that species and parasite stage determination by light microscopy was a very good predictor of infection success, as *P. vivax* with gametocyte infections resulted in approximately 4-fold increased infection success in the mosquitoes as compared to *P. falciparum*. Based on our results we estimate that light microscopy diagnosis before bleeding would enable a further increase of DMFA success rate with *P. vivax* to around 60% if suitable *P. vivax* samples (those with gametocytes by light microscopy) were selected. Similar infectivity rates (45-60%) were measured in *Anopheles aquasalis, Anopheles albitarsis* in Brazilian Amazon, *Anopheles albamanus* in Colombia, *An. dirus* in Thailand and *Anopheles arabiensis* in Ethiopia (Sattabongkot et al., 2003; Solarte et al., 2011; Rios-Velásquez et al., 2013; Vallejo et al., 2016; Tadesse et al., 2018). We did also observe a significant but moderate correlation between *P. vivax* gametocytes and mosquito infection (**Figure 2B**). Other studies observed similar but often stronger positive associations between *P. vivax* gametocyte densities and the proportion of infected mosquitoes in *An. dirus* in Thailand and *An. arabiensis* in Ethiopia (Kiattibutr et al., 2017; Tadesse et al., 2018). However, there are other studies which describe the relationship between *P. vivax* gametocytaemia and mosquito infection as weak with *An. dirus* in Thailand (Sattabongkot et al., 1991; Sattabongkot et al., 2003).

The observed correlation between mosquito infection prevalence and oocyst density was moderate but significant for both *P. falciparum* and *P. vivax* (**Figures 3A–D**). That is, the more mosquitoes are infected during a DMFA, the higher the average number of oocysts in the infected mosquitoes. Our findings is in contrast with a previous study where a strong correlation was observed between the mosquito infection rate and the oocyst rates for *An. dirus* with *P. vivax* (Kiattibutr et al., 2017).

We noted that only 2/45 (4.4%) of samples resulted in mosquito infections from DMFA using blood from the Madang Town Clinic while 36/137 (26.2%) of samples from Yagaum clinic infected mosquitoes. The low infection rate from Madang Town Clinic was mainly because most of the samples were without gametocytes especially *P. vivax* gametocytes. Of the 45 samples 3 samples had only *P. falciparum* gametocytes while 2 had only *P. vivax* gametocytes and one with both *P. falciparum* and *P.vivax* gametocytes. Of the 2 samples that led to successful infections, one had only *P. vivax* gametocytes while the other had both *P.falciparum* and *P. vivax* gametocytes. Another factor which could have influenced the infectivity of the mosquitoes but was not investigated here is impact of temperature fluctuations of the thermal flask while transporting it from Madang to the laboratory, and the longer duration between collection of the sample and the DMFA. It has been shown elsewhere that temperature of thermal flask does influence the infectivity of the mosquitoes (Soumare et al., 2021).

This study provides important insights into the infectivity of symptomatic malaria cases to *An. farauti* in PNG. Overall, we show that symptomatic *P. vivax* infections are more likely to be infectious to mosquitoes as compared to symptomatic *P. falciparum* infections. This may be a result of the differences in gametocyte dynamics that exist between *P. falciparum* and *P. vivax*. We have re-established a DMFA set up in PNG, where frequent access to *P. vivax* infections is provided. This could serve as a platform to test potential transmission blocking vaccines and antimalarials, which act on gametocytes or the mosquito developmental stages of *P. vivax*.

## Data Availability Statement

The raw data supporting the conclusions of this article will be made available by the authors, without undue reservation.

## Ethics Statement

The studies involving human participants were reviewed and approved by the Papua New Guinea Medical Research Advisory Council. Written informed consent to participate in this study was provided by the participants or their legal guardian/next of kin.

## Author Contributions

Designed the study: LR, SK, ML. Conducted the laboratory work: LT, RV, MK, TK, EN. Secured funding: IM, IF, SK. Drafting and preparation of the manuscript: LT, SK. Critically revising the manuscript: SK, RV, MK, TB, LS, IF, LR, CC. All authors contributed to the article and approved the submitted version.

## Funding

This work was supported in part by the Bill and Melinda Gates Foundation (OPP1034577), National Institute of Allergy and Infectious Diseases (NIAID) (5U19AI089686-03), Swiss National Science Foundation (310030_134889), and National Health and Medical Research Council (NHMRC) of Australia (GNT1127356). LT is supported by a PhD scholarship from James Cook University. IM is supported by a Research Fellowship from NHMRC. LR and SK are supported by Career Development Fellowships from NHMRC of Australia.

## Conflict of Interest

The authors declare that the research was conducted in the absence of any commercial or financial relationships that could be construed as a potential conflict of interest.

## Publisher’s Note

All claims expressed in this article are solely those of the authors and do not necessarily represent those of their affiliated organizations, or those of the publisher, the editors and the reviewers. Any product that may be evaluated in this article, or claim that may be made by its manufacturer, is not guaranteed or endorsed by the publisher.

## Abbreviations

DMFA, Direct membrane feeding assays; *P.vivax*, *Plasmodium vivax*; *P. falciparum*, *Plasmodium falciparum*; *An. Farauti*, *Anopheles farauti*; RDT, Rapid diagnostic kits; HRP2, Histidine rich protein 2; pLDH, Parasite lactate dehydrogenase; qPCR, Quantitative real time polymerase chain reaction assay.
