# Supplementary material for: Infectivity of Symptomatic Malaria Patients to Anopheles farauti Colony Mosquitoes in Papua New Guinea
Source: Front Cell Infect Microbiol. 2021 Dec 22;11:771233. doi: 10.3389/fcimb.2021.771233 (PMC8729879; doi:10.3389/fcimb.2021.771233)
Supplement: Supplementary file 1 [file Table_1.docx]

**Supplementary Table 1**. Showing the raw data of the location, the diagnostic methods and the successful mosquito infections

| **Location^Ø^** | **RDT** | **Pf asex (/µl)** | **Pf gameto (/µl)** | **Pv asex (/µl)** | **Pv gameto (/µl)** | **Pf qPCR** | **Pv qPCR** | **Total no. of mos. disected** | **Total no. mos. with oocyst/ sprozoites** | **Total no of mos. without oocyst/ sprozoites** | **Mos. infection rate** | **Total oocyst/ sprozoite Count** | **Mean oocysts/ sprozoite per mos.** |
| --- | --- | --- | --- | --- | --- | --- | --- | --- | --- | --- | --- | --- | --- |
| Town | PLDH | 0 | 0 | 1503 | 47 | 0.00E+00 | 1.90E+02 | 52 | 15 | 37 | 28.8 | 140 | 9 |
| Town | HRP2 | 0 | 6810 | 0 | 0 | 2.95E+01 | 0.00E+00 | 44 | 31 | 10 | 70.5 | <226 300 | <7300^*^ |
| Yagaum | Mixed^§^ | 1824 | 1558 | 0 | 0 | 1.20E+02 | 2.75E+03 | 24 | 2 | 22 | 8.3 | <10 000 | <5000^*^ |
| Yagaum | PLDH | 0 | 0 | 507 | 165 | 3.46E+03 | 2.69E+03 | 79 | 8 | 71 | 10.1 | <20 000 | <2500^*^ |
| Yagaum | Mixed | 51040 | 16620 | 0 | 0 | 3.98E+00 | 0.00E+00 | 29 | 14 | 15 | 48.3 | <80 000 | <5714^*^ |
| Yagaum | Mixed | 0 | 0 | 1344 | 0 | 0.00E+00 | 0.00E+00 | 55 | 2 | 53 | 3.6 | 2 | 1 |
| Yagaum | Mixed | 1540 | 0 | 0 | 0 | 7.49E+02 | 0.00E+00 | 88 | 4 | 85 | 4.5 | 9 | 2 |
| Yagaum | HRP2 | 0 | 0 | 272 | 32 | 0.00E+00 | 4.28E+02 | 113 | 101 | 12 | 89 | 770 | 8 |
| Yagaum | PLDH | 110 | 0 | 1373 | 47 | 0.00E+00 | 3.57E+02 | 89 | 83 | 6 | 93.3 | 569 | 7 |
| Yagaum | PLDH | 0 | 0 | 12211 | 0 | 0.00E+00 | 2.80E+00 | 113 | 43 | 70 | 38 | 118 | 3 |
| Yagaum | PLDH | 0 | 0 | 0 | 0 | 0.00E+00 | 0.00E+00 | 14 | 4 | 10 | 28.6 | 50 | 13 |
| Yagaum | PLDH | 0 | 0 | 11680 | 0 | 0.00E+00 | 2.13E+04 | 132 | 22 | 110 | 17 | 62 | 3 |
| Yagaum | HRP2 | 0 | 0 | 2418 | 0 | 0.00E+00 | 2.98E+01 | 102 | 7 | 95 | 7 | 11 | 2 |
| Yagaum | HRP2 | 0 | 0 | 966 | 0 | 0.00E+00 | 2.98E+01 | 71 | 54 | 17 | 76 | 204 | 4 |
| Yagaum | HRP2 | 0 | 0 | 9013 | 198 | 2.89E+01 | 3.04E+00 | 93 | 84 | 9 | 90 | 1167 | 14 |
| Yagaum | HRP2 | 0 | 0 | 7324 | 217 | 1.94E+03 | 0.00E+00 | 128 | 56 | 72 | 44 | 310 | 6 |
| Yagaum | HRP2 | 0 | 0 | 136 | 0 | 0.00E+00 | 5.12E+02 | 30 | 8 | 22 | 26.7 | 55 | 7 |
| Yagaum | HRP2 | 0 | 0 | 4240 | 0 | 0.00E+00 | 3.04E+00 | 52 | 4 | 48 | 8 | 20 | 5 |
| Yagaum | HRP2 | 0 | 0 | 0 | 0 | 0.00E+00 | 5.79E+02 | 24 | 9 | 15 | 37.5 | 48 | 5 |
| Yagaum | Mixed | 8960 | 0 | 0 | 0 | 2.56E+04 | 0.00E+00 | 37 | 21 | 16 | 56.8 | 121 | 6 |
| Yagaum | HRP2 | 0 | 0 | 6667 | 137 | 0.00E+00 | 2.19E+07 | 57 | 33 | 24 | 58 | 202 | 6 |
| Yagaum | Mixed | 0 | 0 | 540 | 111 | 0.00E+00 | 3.85E+00 | 27 | 5 | 22 | 19 | 26 | 5 |
| Yagaum | HRP2 | 7251 | 0 | 0 | 0 | 0.00E+00 | 4.82E+00 | 41 | 30 | 11 | 73.2 | 170 | 6 |
| Yagaum | HRP2 | 0 | 0 | 10540 | 500 | 0.00E+00 | 1.75E+01 | 127 | 119 | 8 | 94 | 1377 | 12 |
| Yagaum | HRP2 | 0 | 0 | 1197 | 71 | 0.00E+00 | 24.07760 | 39 | 17 | 22 | 44 | 68 | 4 |
| Yagaum | Mixed | 9899 | 0 | 0 | 0 | 9.87E+01 | 0.00E+00 | 36 | 2 | 34 | 5.6 | 4 | 2 |
| Yagaum | PLDH | 0 | 0 | 304 | 0 | 0.00E+00 | 1.91E+00 | 48 | 4 | 44 | 8.3 | 6 | 2 |
| Yagaum | Mixed | 4640 | 0 | 0 | 0 | 5.06E+00 | 0.00E+00 | 59 | 4 | 55 | 6.8 | 8 | 2 |
| Yagaum | Mixed | 5594 | 0 | 0 | 0 | 2.63E+04 | 0.00E+00 | 63 | 3 | 60 | 4.8 | 4 | 1 |
| Yagaum | Mixed | 12164 | 0 | 0 | 0 | 2.93E+02 | 8.47E+00 | 52 | 2 | 50 | 3.8 | 3 | 2 |
| Yagaum | HRP2 | 576 | 40 | 0 | 24 | 0.00E+00 | 1.99E+02 | 29 | 3 | 26 | 10.3 | 7 | 2 |
| Yagaum | HRP2 | 0 | 0 | 28109 | 177 | 0.00E+00 | 6.95E+00 | 19 | 8 | 11 | 42 | 19 | 2 |
| Yagaum | PLDH | 0 | 0 | 10731 | 699 | 0.00E+00 | 6.23E+01 | 42 | 31 | 11 | 74 | 137 | 4 |
| Yagaum | PLDH | 0 | 0 | 4654 | 60 | 0.00E+00 | 2.59E+06 | 95 | 4 | 91 | 4 | 8 | 2 |
| Yagaum | PLDH | 44557 | 239 | 4926 | 199 | 0.00E+00 | 3.56E+06 | 40 | 10 | 30 | 25.0 | 37 | 4 |
| Yagaum | PLDH | 0 | 0 | 10280 | 0 | 0.00E+00 | 51598853.68 | 79 | 73 | 6 | 92 | 1545 | 21 |
| Yagaum | PLDH | 0 | 0 | 576 | 64 | 0.00E+00 | 3.36E+02 | 39 | 13 | 26 | 33 | 47 | 4 |
| Yagaum | PLDH | 0 | 0 | 32480 | 840 | 0.00E+00 | 5.68E+01 | 41 | 39 | 2 | 95 | 6569 | 168 |
|  |  |  |  |  |  |  |  |  |  |  |  |  |  |
|  |  |  |  |  |  |  |  |  |  |  |  |  |  |

^*^These successful infections were only dissected for sporozoites ^§^ Mixed: both HRP2 and pLDH **^Ø^**Madang Town Clinic, Yagaum Hospital Pf: *Plasmodium falciparum*, Pv: *Plasmodium vivax*, asex: asexual, gameto: gametocytes, mos: mosquitos, no: number
